# Exogenous interleukin 33 enhances the brain’s lymphatic drainage and toxic protein clearance in acute traumatic brain injury mice

**DOI:** 10.1186/s40478-023-01555-4

**Published:** 2023-04-07

**Authors:** Mingqi Liu, Jinhao Huang, Tao Liu, Jiangyuan Yuan, Chuanxiang Lv, Zhuang Sha, Chenrui Wu, Weiwei Jiang, Xuanhui Liu, Meng Nie, Yupeng Chen, Shiying Dong, Yu Qian, Chuang Gao, Yibing Fan, Di Wu, Rongcai Jiang

**Affiliations:** 1 Department of Neurosurgery, Tianjin Medical University General Hospital, 154 Anshan Road, Tianjin, 300052 China; 2 Tianjin Neurological Institute, Key Laboratory of Post Neuro-Injury Neuro-Repair and Regeneration in Central Nervous System, Ministry of Education and Tianjin City, Tianjin, 300052 China; 3 Department of Neurosurgery, The First Clinical Hospital, Jilin University, Changchun, China; 4 Department of Neurosurgery, Tianjin First Central Hospital, Tianjin, China

**Keywords:** Traumatic brain injury, Interleukin 33, Glymphatic system, Aquaporin-4, Meningeal lymphatic vessels

## Abstract

The persistent dysregulation and accumulation of poisonous proteins from destructive neural tissues and cells activate pathological mechanisms after traumatic brain injury (TBI). The lymphatic drainage system of the brain, composed of the glymphatic system and meningeal lymphatic vessels (MLVs), plays an essential role in the clearance of toxic waste after brain injury. The neuroprotective effect of interleukin 33 (IL-33) in TBI mice has been demonstrated; however, its impact on brain lymphatic drainage is unclear. Here, we established a fluid percussion injury model to examine the IL-33 administration effects on neurological function and lymphatic drainage in the acute brain of TBI mice. We verified that exogenous IL-33 could improve the motor and memory skills of TBI mice and demonstrated that in the acute phase, it increased the exchange of cerebrospinal and interstitial fluid, reversed the dysregulation and depolarization of aquaporin-4 in the cortex and hippocampus, improved the drainage of MLVs to deep cervical lymph nodes, and reduced tau accumulation and glial activation. We speculate that the protective effect of exogenous IL-33 on TBI mice’s motor and cognitive functions is related to the enhancement of brain lymphatic drainage and toxic metabolite clearance from the cortex and hippocampus in the acute stage. These data further support the notion that IL-33 therapy may be an effective treatment strategy for alleviating acute brain injury after TBI.

## Introduction

Traumatic brain injury (TBI) is a severe burden to the health system owing to its high mortality and disability rates. According to statistics, approximately 69 million people experience TBI for various reasons annually [15]. It is estimated that even in patients with mild TBI, 15–30% of them will experience long-term sequelae of exercise and cognitive defects after exposure [24]. Moderate to severe TBI is an established risk factor for neurodegenerative diseases, including Alzheimer’s disease (AD) [23, 57]. The occurrence of inflammatory events, persistent protein dysregulation, and accumulation (including tau) are considered to be active pathological mechanisms in TBI’s acute and long-term consequences [4, 6]. Tau phosphorylation can occur as early as 12 h after TBI and rapidly spreads to other neurons, causing apoptosis [39]. However, to date, there are no recognized and effective drugs or measures to improve TBI’s short- and long-term prognoses.

The glymphatic system, which has recently received widespread attention, is an aquaporin-4 (AQP4)-dependent perivascular channel formed by astrocytes. It can effectively clear metabolic waste from the central nervous system (CNS) and is influenced by sleep, circadian rhythm, arterial pulsation, and other factors [25, 32, 73]. Glymphatic dysfunction has been demonstrated in rodent models, such as not limited to TBI, AD, and multiple sclerosis (MS), and is probably related to the disturbance of aquaporin-4 (AQP4) expression [17, 27, 30]. Iliff et al. [30, 59] reported that functional deficits in the glymphatic system, loss of perivascular AQP4 localization, and reactive hyperplasia of astrocytes were observed at an early stage after TBI (1-3D). Additionally, as a connection pathway between glymphatic and cervical lymph nodes (CLN), meningeal lymphatic vessels (MLVs) and their close relationship with glymphatic have attracted extensive attention worldwide [48]. Ishida et al. [33] found impaired MLVs function in AQP4-KO mice, manifested as reduced intracisternal tracer drainage to the deep CLN (dCLN). Treatment of mice with vascular endothelial growth factor C (VEGFC), which promotes MLVs proliferation and migration, alleviates TBI-driven MLVs dysfunction, inflammation, and neurological deficits [5, 44]. These findings indicate a close relationship between TBI and the lymphatic drainage systems in the brain.

Interleukin-33 (IL-33) is a tissue-derived cytokine from the IL-1 family that localizes to the cell nucleus but is also functional when released as a cell-free cytokine. It is expressed abundantly in the CNS, mainly in astrocytes, and to a lesser extent in oligodendrocytes, microglia, and neurons [63]. IL-33 can be released by necrotic cells following tissue damage as an endogenous danger signal or “alarmin” [45], binding to the ST2 protein (the only well-documented receptor for IL-33) to play a complex immunomodulatory role in rodent models of neurological diseases, such as AD, MS, and stroke [18, 38, 40]. ST2 exists primarily in two forms: a membrane-bound form, which is distributed on the surface of various immune cells and binds to IL-33, and a soluble form (sST2), which acts as a decoy receptor that blocks IL-33 signaling [45]. Exogenous IL-33 can regulate autophagy, endoplasmic reticulum stress, apoptosis, and regulatory T cell (Treg) responses via the IL-33/ST2 signaling pathway to improve the prognosis of TBI rodent models [22, 72]. Recently, it was reported that IL-33-KO mice develop chronic neurodegeneration and AD-like dementia in late life, and intraperitoneal injection of recombinant IL-33 in IL-33-KO mice induces robust expression of AQP4 in the perivascular astrocyte endfeet and accelerates the drainage of intracerebroventricularly injected peptides [70]. Moreover, another recent clinical study indicated an association between high plasma levels of IL-33 and cognitive preservation in patients with mild amnestic cognitive impairment and AD [43]. Therefore, we hypothesized that IL-33 might alleviate neurological deficits after TBI by regulating lymphatic drainage in the brain. Here, we administered exogenous IL-33 to TBI mice across the blood–brain barrier, set sST2 treatment as a control group, and assessed neurological function and brain lymphatic drainage changes.

## Materials and methods

### Animals

Here, 8–10 week-old male and female C57BL/6 mice (Beijing Vital River Laboratory Animal Technology Inc., China) were housed in specific pathogen-free conditions under a standard 12 h light/dark cycle with food and water ad libitum. Mice were subjected to brief anesthesia induced by 2-4% isoflurane, followed by the maintenance of 1.5% isoflurane in oxygen-enriched air (20% oxygen/80% air) while allowing for spontaneous ventilation throughout the investigation. All experiments were approved by the Animal Care and Use Committee of Tianjin Medical University General Hospital, China, and were conducted following the ARRIVE guidelines. Appropriate measures were observed to ensure minimal pain and discomfort in the animals.

### Drug administration and experimental design

The experimental groups were as follows: sham group (identical surgical procedures except for brain injury + saline), TBI group, TBI + IL-33 group, and TBI + sST2 group. IL-33 (20 ng/μl, 5 μl, AF3626, R&D systems, USA), sST2 (20 ng/μl, 5 μl, AF1004, R&D systems, USA), or an equivalent volume of sterile phosphate-buffered saline (PBS) was administered intracisternal magna (I.C.M) 60 min before TBI.

### Fluid percussion injury (FPI) model

The FPI model was based on a previous study [76]. Initially, anesthetized mice underwent a 3 mm craniotomy 2 mm posterior to the bregma and 1.5 mm lateral to the sagittal suture. Subsequently, a brief fluid pressure pulse was applied to the intact dura through the bone window using a fluid percussion device (Custom Design & Fabrication, Richmond, VA, USA) filled with sterile saline; the pressure was controlled at 1.9 ± 0.2 atm. Next, the scalp incision was closed with sutures, and the mice were placed on a heated pad at 37 °C to maintain normal temperature until awakening. Except for the strike program, the sham group underwent the same procedure.

### Modified neurological severity score (mNSS)

The mNSS, which includes a composite of motor, sensory, reflex, and balance tests [10], was used to measure neurological deficits on 1D, 3D, 5D, 7D, and 14D after TBI. The mNSS assesses neurological functioning on a scale of 0–18. The higher the overall composite score, the worse the functional impairment.

### Rotarod test

Mouse motor coordination and balance alterations were assessed using a rotarod apparatus (YLS-4C, Beijing) on 1D, 3D, 5D, 7D, and 14D after TBI, as previously described [20]. Before the trial, all mice were trained on the rotarod (5–10 RPM) for 300 s. On each testing day, the rod was conducted at a uniform accelerating speed from 5 to 40 RPM for 300 s, repeated three times. The latency to fall of each mouse was recorded and averaged.

### Novel object recognition test

The novel object recognition test was modified based on previous studies [64]. Briefly, the tests were performed in a 40 × 40 × 40 cm arena in a quiet room. During the habituation session on the first day, each mouse was allowed to explore the arena for 5 min. The next day, the mice explored the same arena for 5 min, with two identical objects placed equidistant from the sidewall. Following a retention delay of 4 h, the mice were tested for short-term memory of the objects. During the test session, mice were allowed to explore the arena for 5 min, with one of the old objects replaced by a new object. Exploration times were recorded and analyzed using a video tracking system (EthoVision XT 13, Noldus Information Technology, Wageningen, Netherlands). The discrimination index was calculated as follows: novel exploration time/total exploration time.

### Morris water maze test

From the 15 to 20th days after TBI, the mice’ spatial learning and memory abilities were assessed using the Morris water maze test [20]. Briefly, the experiment consisted of a training phase of 5 consecutive days and a testing phase of 1 day. For training on the 15–19th days, the mice were trained to seek a hidden platform within 90 s and stay on the platform for 15 s. If the platform was not found within 90 s, the mouse was gently guided to reach the platform and remained there for 15 s, and the recording latency was 90 s. During the training phase, the mice were trained from the first to the fourth quadrant four times a day. The test was conducted on the 20th day, with the platform removed, and the mouse was placed on the opposite side of the platform quadrant. We used video tracking software (EthoVision XT 13, Noldus Information Technology, Wageningen, Netherlands) to record and analyze the latency to the platform, platform crossing times, and swimming traces.

### Intracisternal and intrahippocampal injections

Cerebrospinal fluid (CSF) tracer injections were performed as described [36, 71].

*Intracisternal injection* After the mouse was anesthetized, the posterior neck hair was shaved, the head was fixed on a stereotactic frame, and the skin was incised at the midline to expose the posterior atlanto-occipital membrane that covered the cisterna. Using a 25-μl syringe (#710 RN, 0.485 mm ID; Hamilton) and 33 g needle (15 mm, pst 4–12, Hamilton), 5 μl of 2.5% 70 KD tracer Rhodamine B isothiocyanate dextran (RITC-Dextran, R9379, Sigma) was injected at a rate of 1 μl/min. The tracer was allowed to circulate for 60 min after injection. The needle inserted into the cisterna was retained throughout the injection until the end of the cycle to prevent tracer leakage and backflow when the needle was removed. Subsequently, the mice were euthanized and perfused with cold PBS (Solarbio), and the tissues were collected and fixed in 4% paraformaldehyde (Solarbio) for 12 h. Fixed brains were sectioned into coronal sections with a thickness of 100 μm using a cryostat microtome (CM1950, Leica Biosystems, Nußloch, Germany) and observed under an inverted fluorescence microscope (IX73, Olympus).

*Intrahippocampal injection* The previous treatment of mice was the same as that used for the intracisternal injection. Use a 5 μl syringe (#75RN, 0.343 mm ID, Hamilton) and a 33 g needle (15 mm, pst3, Hamilton) injected 0.4 μl 2.5% 70 KD RITC-Dextran tracer into the hippocampus at a rate of 0.2 μl/min (− 2.0 mm anterior–posterior, 1.5 mm medial–lateral, and − 2 mm dorsal–ventral relative to the bregma). After injection, the needle was allowed to stay at the injection site for 5 min and then withdrawn slowly to prevent the solution from leaking. The tracer was circulated for an additional 60 min after the end of injection, and the subsequent treatment of mice was comparable to that of the intracisternal injection.

### Immunofluorescence staining

The meninges, brain slices, and dCLN were obtained for staining 3 days after injury. For the meningeal whole-mount specimen collection, we stripped the skin and muscle from the skull, removed the cranium and skull base with surgical scissors, and fixed overnight at 4 °C in 4% paraformaldehyde. Meninges (dura and arachnoid) were carefully dissected from the skull. However, only the dorsal and occipital meninges remained intact. Whole-mount meninges were transferred to PBS for further immunofluorescence staining. For slice collection of brains and dCLN, they were first removed and fixed overnight at 4 °C in 4% paraformaldehyde, then they were dehydrated using 15% and 30% sucrose solutions. After that, the brains and dCLN were immersed in an optimal cutting temperature compound (Sakura Finetek USA, Torrance, CA, USA) and cut into 8-μm coronal sections using a cryostat microtome (CM1950, Leica Biosystems, Nußloch, Germany). Meninges and tissue sections were washed in PBS and blocked with 3% bovine serum albumin, 0.2% Triton, and 0.05% Tween 20 in PBS for 1.5 h at room temperature. The primary and secondary antibodies were incubated overnight at 4 °C and for 2 h at room temperature, respectively. The primary antibodies used were as follows: rabbit anti- LYVE-1 (1:200, Abcam, ab14917), rabbit anti-AQP4 (1:500, Cell Signaling Technology, 59678), goat anti-CD31 (1:200, R&D Systems, AF3628), chicken anti-GFAP (1:500, Abcam, ab4674), goat anti-IBA1(1:200, Abcam, ab5076), and rabbit anti-S100β (1:100, Abcam, ab52642). The stained sections were observed under an inverted fluorescence microscope (IX73, Olympus).

### Western blotting

Protein levels in the cortex, hippocampus, and meninges were detected by western blotting using a standard method described previously [47]. Briefly, the tissues were collected 3 days after TBI, following euthanasia and perfusion with cold PBS. The total protein in the tissues of each group was extracted using radio-immunoprecipitation assay lysis buffer containing protease and phosphatase inhibitors (P1260 Solarbio, Beijing, China), loaded on 10% or 12% SDS-PAGE gel with the constant current; subsequently, it was transferred to a polyvinylidene difluoride (PVDF) membrane using a wet electrotransfer device (Bio-Rad). The following specific primary antibodies were incubated with PVDF membranes overnight at 4 °C: rabbit anti-VEGF receptor 3 (VEGFR3) (1:500, ThermoFisher, PA5-16871), rabbit anti-vascular endothelial (VE)-cadherin (1:500, ThermoFisher, 36-1900), rabbit anti-PROX1 (1:500, Abclonal, A9047), rabbit anti-FOXC2 (1:500, Proteintech, 23066–1-ap), rabbit anti-VEGFC (1:500, Abclonal, A2556), mouse anti-GAPDH (1:1000, ZSGB-BIO, TA-08), mouse anti-Tau5 (1:1000, Abcam, ab80579), rabbit anti-phospho-tau (Thr205) (1:2000, Abclonal, AP0168), rabbit anti-phospho-tau (Ser404) (1:2000, Abclonal, AP0170), rabbit anti-phospho-tau (Ser396) (1:1000, Cell Signaling Technology, 9632 s). The membranes were washed three times after incubation. After incubation with horseradish peroxidase-conjugated secondary antibodies, protein bands were visualized using an enhanced chemiluminescence system (Millipore Sigma). A density analysis of the bands was performed using the FIJI (ImageJ) software.

### Image and statistical analysis

All image analyses were performed using FIJI and Origin 9.1 software. All statistical analyses were performed using the GraphPad Prism software (version 8.0). A uniform linear adjustment of pixel intensity was applied to the images shown in the figures. All immunofluorescent picture analyses, including AQP4 localization around macrovessels and microvessels, were quantified using FIJI. Briefly, subjective values, including regions of interest and uniform threshold selection, were identified in a blinded manual fashion. Origin was used to map AQP4 immunofluorescence projections across large cortical vessels. Fluorescence intensities of AQP4 markers were measured on a single 40 μm axis perpendicular to the vessel direction, generating a linear fluorescence plot extending from the brain tissue to the vessels and again to the surrounding brain tissue. AQP4 perivascular polarization was measured as previously described [27, 41]. Briefly, median immunofluorescence intensity in the perivascular region was measured. Threshold analysis was used to measure the percentage of areas with AQP4 immunofluorescence greater than or equal to perivascular AQP4 immunofluorescence (AQP4% area). Polarization was expressed as the percentage of areas with lower AQP4 immunoreactivity than the perivascular endfeet (1–AQP4% area). The CD31-positive area was defined as the perivascular region. Capillaries were defined as vessels < 10 μm, and large vessels were defined as vessels > 10 μm.

The sample size in this study was similar to that reported in previous studies. One-way or two-way analyses of variance were used to determine statistical differences in studies involving two or more groups with one or more characteristics. Tukey’s post hoc test was used for multiple comparisons. Statistical significance was set at *p* < 0.05. Unless otherwise specified, all data are presented as the mean ± standard deviation.

## Results

### Exogenous IL-33 improved motor and cognitive function in TBI mice

**Fig. 1:**
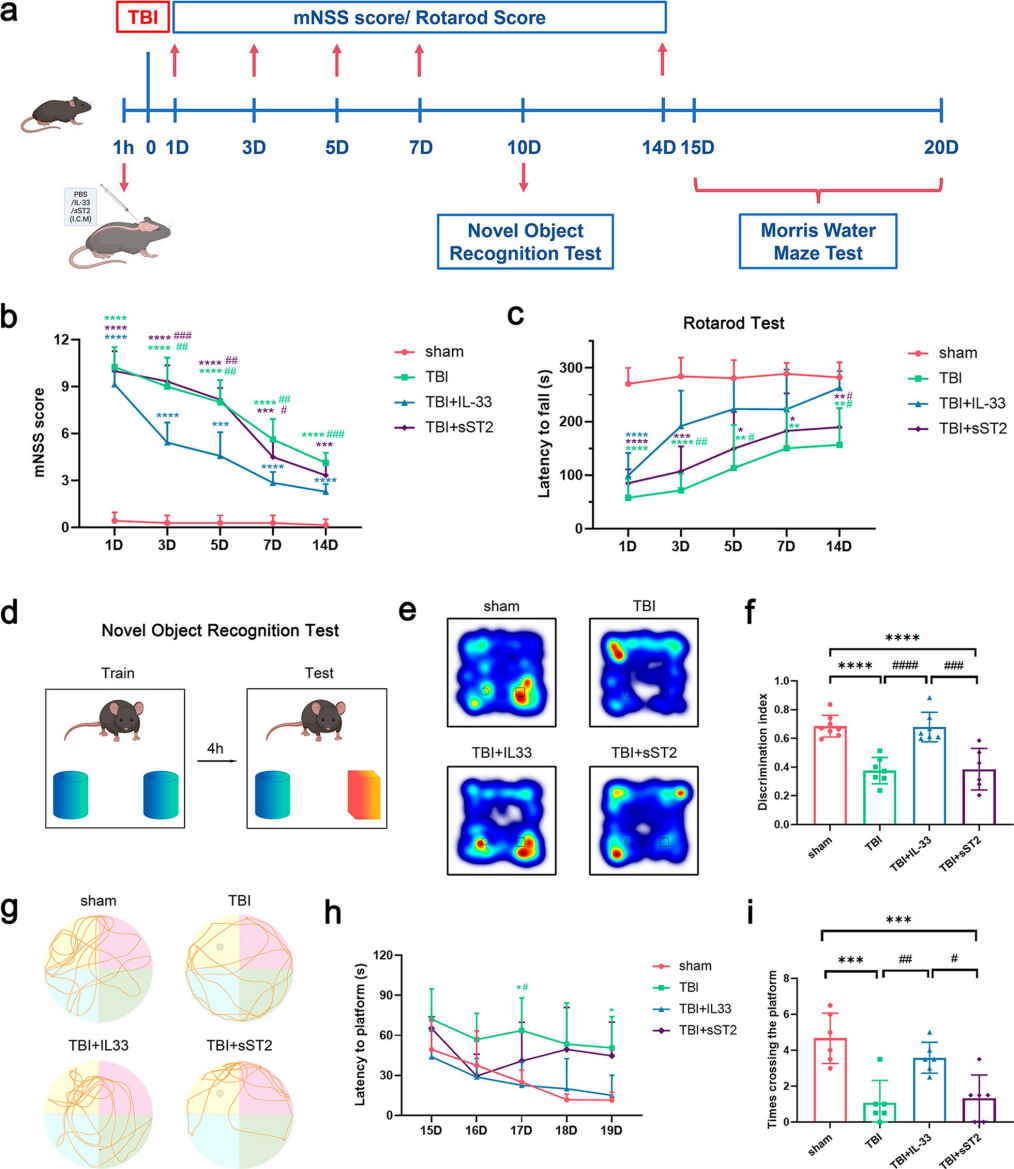
The effect of IL-33/sST2 treatment on the neurological function of acute TBI mice. **a** the experimental timeline of IL-33/sST2 treatment and behavioral tests. motor function is assessed by mNSS score (**b**) and rotarod test (**c**). **d** Schematic of the novel object recognition test. Cylinders and cuboids indicate different objects. **e** Representative heatmaps of animal tracking following novel object recognition test. **f** The discrimination index for each group. **g** Representative track sheets during the probe trial of the Morris water maze test (last 60 s). **h** the learning curve during the training phase. **I** Latency of mice to examine the platform. All data are presented as mean ± SD (n = 6–8 per group). *p < 0.05 versus sham group, **p < 0.01 versus sham group, ***p < 0.001 versus sham group, ****p < 0.0001 versus sham group, #p < 0.05 versus TBI + IL-33 group, ##*p* < 0.01 versus TBI + IL-33 group, ###*p* < 0.001 versus TBI + IL-33 group, ####*p* < 0.0001 versus TBI + IL-33 group

It has been reported that IL-33 treatment could improve the neurological function of TBI mice [22, 72]. We created a FPI model to evaluate the effect of IL-33 on the prognosis of TBI in mice. The mNSS and rotarod tests were used to evaluate motor function. The new object recognition and Morris water maze tests were used to assess the cognitive function of the mice after injury (Fig. 1a). The results showed that motor function was impaired 1 day after the injury. The motor function of the IL-33 treatment group was significantly improved on days 3, 5, 7, and 14 (Fig. 1b–c). The cognitive tests showed similar results, with exogenous IL-33 significantly alleviating TBI-induced deficits in short-term and spatial memory (Fig. 1d–i). The neurological function examination revealed no significant difference between the sST2 treatment and non-treatment groups.

### Exogenous IL-33 enhanced the function of the glymphatic system solute drainage and improved the perturbed expression of perivascular AQP4 in the cortex and hippocampus of acute TBI mice

**Fig. 2:**
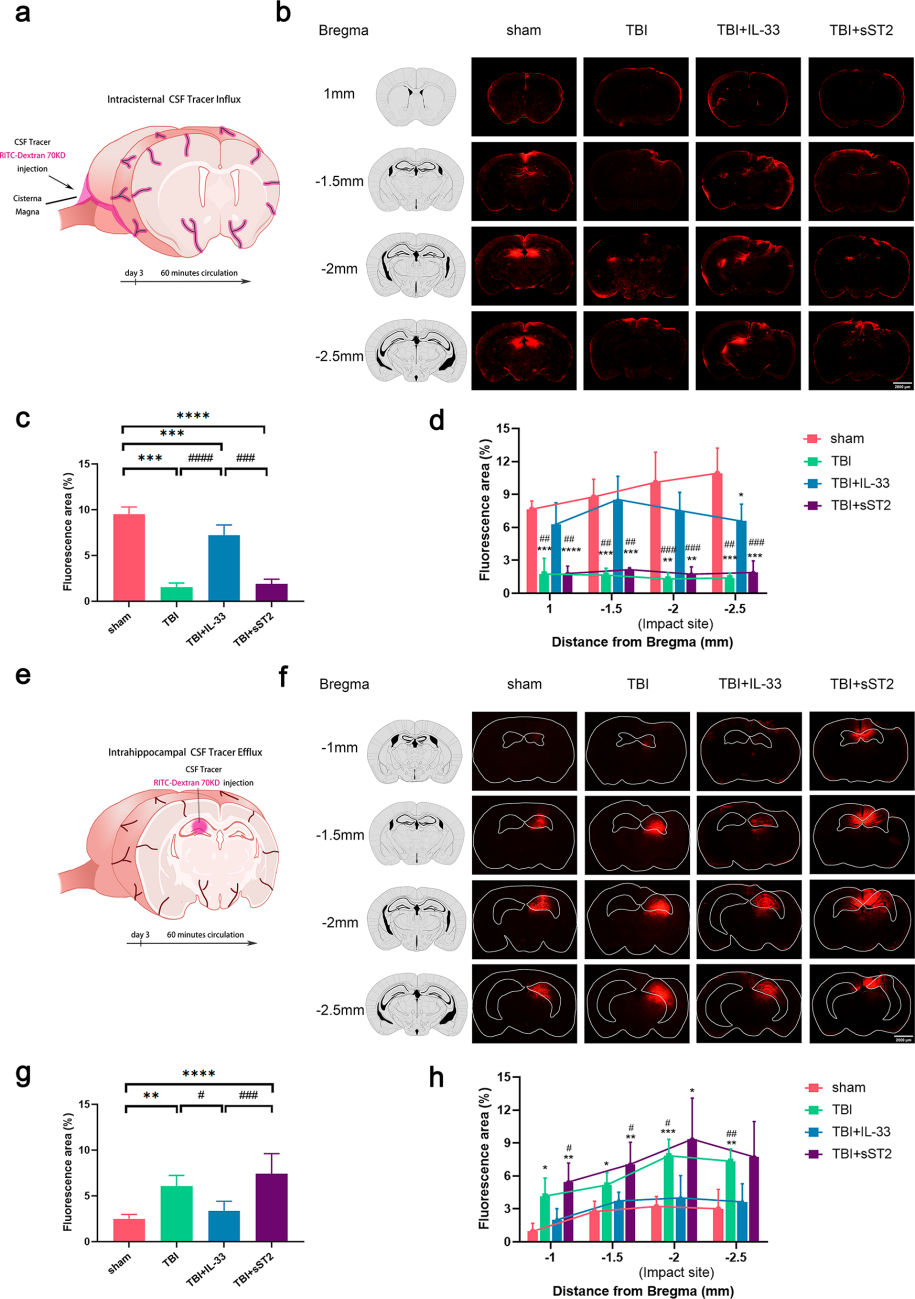
Altered glymphatic intracisternal solute influx and intrahippocampal solute efflux of acute TBI mice. **a** CSF tracer (RITC-Dextran, 70 KD) was injected intracisternally into acute TBI mice, and perfusion was fixed after 60 min of circulation. **b** Representative images of RITC-Dextran accumulation in four coronal brain sections at bregma + 1.0 mm, − 1.5 mm, − 2 mm, and − 2.5 mm. **c** Average quantification of percent area covered by RITC-Dextran. Four slices per animal were analyzed. **d** Quantification of percent area covered by the tracers per slice from different distances to the bregma. **e** RITC-Dextran was injected into the hippocampus of acute TBI mice. **f** Representative images of RITC-Dextran clearance in four coronal brain sections at bregma − 1.0 mm, − 1.5 mm, − 2 mm, and − 2.5 mm. **g**–**h** Quantification of percent area covered by the tracers for mean and per section. All data are presented as mean ± SD (n = 6 per group). *p < 0.05 versus sham group, **p < 0.01 versus sham group, ***p < 0.001 versus sham group, ****p < 0.0001 verus sham group, #*p* < 0.05 versus TBI + IL-33 group, ##*p* < 0.01 versus TBI + IL-33 group, ###*p* < 0.001 versus TBI + IL-33 group, ####*p* < 0.0001 versus TBI + IL-33 group

To evaluate the effect of exogenous IL-33 on the function of the glymphatic system in acute TBI mice, we injected 70 KD tracer RITC-dextran into the mouse’s cisterna magna 3 days after injury (Fig. 2a). After 60 min of circulation, whole-brain sections were collected. The inflow of the tracer into the brain parenchyma in the IL-33 treatment group was significantly greater than that in the non-treatment and sST2 treatment groups (Fig. 2b–d). We also observed that tracer accumulation in the hippocampus was more pronounced than that in other areas (Fig. 2b). Subsequently, we evaluated whether IL-33 alters the hippocampal solute clearance rate in TBI mice. We conducted an intrahippocampal tracer injection experiment at the same timeline as described above (Fig. 2e). After 60 min of cycling and sample processing, representative sections were analyzed to comprehensively assess hippocampal tracer residues (Fig. 2f). The tracer residue in the IL-33 treatment group was significantly lower than that in the untreated group (Fig. 2g–h). Furthermore, the sST2 treatment group showed a worse tracer drainage trend than the non-treatment group, although the difference was not statistically significant.

**Fig. 3:**
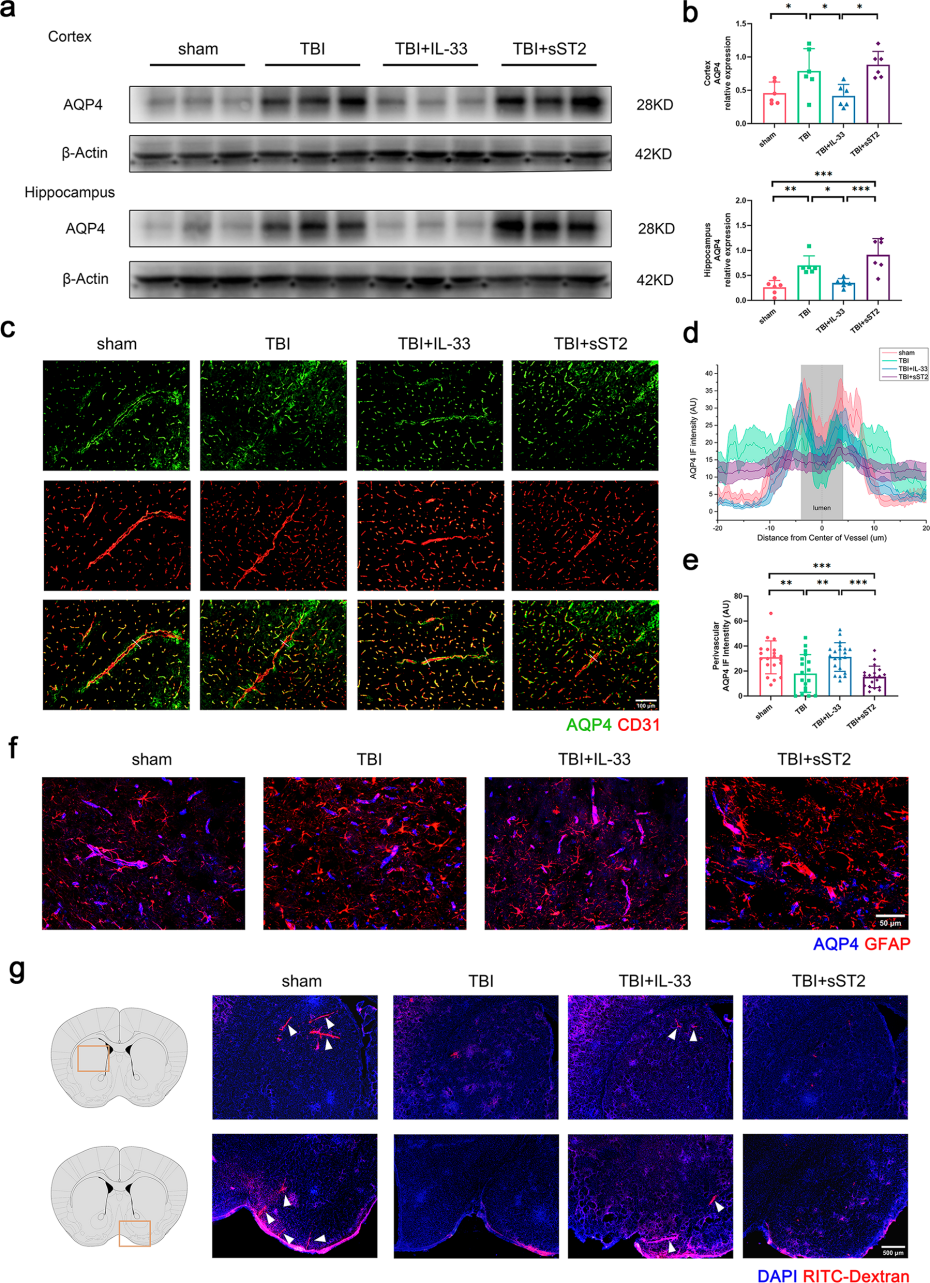
AQP4 expression and perivascular immunofluorescence analysis for large vessels of acute TBI mice. Western blot scans (**a**) and quantification (**b**) of AQP4 protein in the cortex and hippocampus. **c** Representative immunofluorescence images show large vessels in the cortex stained for AQP4 and CD31. White lines indicate the placement of 40-μm axis perpendicular to the blood vessels for quantifying expression across the vessel cross-sections. **d** AQP4 immunofluorescence projections across large cortical vessels were quantified (18–21 vessels from 6 animals per group). Graph showing solid lines for mean values with SEM shown as shading. **e** Quantification of AQP4 expression surrounding large cortical vessels (0–1 μm from the vessel wall). **f** Representative cortex sections stained for AQP4 and GFAP, illustrating altered astrocyte endfeet AQP4 coverage of acute TBI mice. **g** Representative images show CSF tracer penetrating brain parenchyma along large vessels (arrows) to different degrees. All data are presented as mean ± SD (n = 6 per group).*p < 0.05, **p < 0.01, ***p < 0.001

First, to explore how exogenous IL-33 affects the function of the glymphatic system, we analyzed the content of AQP4 protein in the mouse cortex and hippocampus 3 days after TBI, showing that AQP4 protein increased after TBI. Simultaneously, it was significantly decreased in the IL-33 treatment group. No difference was observed between the sST2 treatment and non-treatment groups (Fig. 3a–b).

Subsequently, we analyzed the distribution and fluorescence intensity of AQP4 in the cortex of acute TBI mice near large vessels (diameter > 10 μm) in the cortex of acute TBI mice. Representative images showed an increase in diffuse AQP4 fluorescence in the cortex, whereas IL-33 could reconcentrate AQP4 in the periphery of CD31-labeled vessels (Fig. 3c). The projection of AQP4 fluorescence could more clearly show that AQP4 in the IL-33 treatment group was concentrated on both sides of the vessel wall (the gray shadow represents the vessel lumen) (Fig. 3d). Correspondingly, the quantification of AQP4 intensity was performed in the perivascular area (0–1 μm from the vessel wall) of the cortex (Fig. 3e). However, no significant difference was observed between the sST2 treatment and non-treatment groups.

Typical immunofluorescence images double-stained for AQP4 and GFAP demonstrated reduced astrocytic expression of AQP4 in the cortex of TBI mice. The IL-33 treatment group showed an increase in endfoot AQP4 vessel coverage compared with the non-treatment and sST2 treatment groups (Fig. 3f). Consistent with this, the images of whole brain sections intracisternally injected with the tracer also fully demonstrated the different degrees of infiltration of the CSF tracer along the penetrating vasculature of the brain parenchyma in each group (Fig. 3g).

**Fig. 4:**
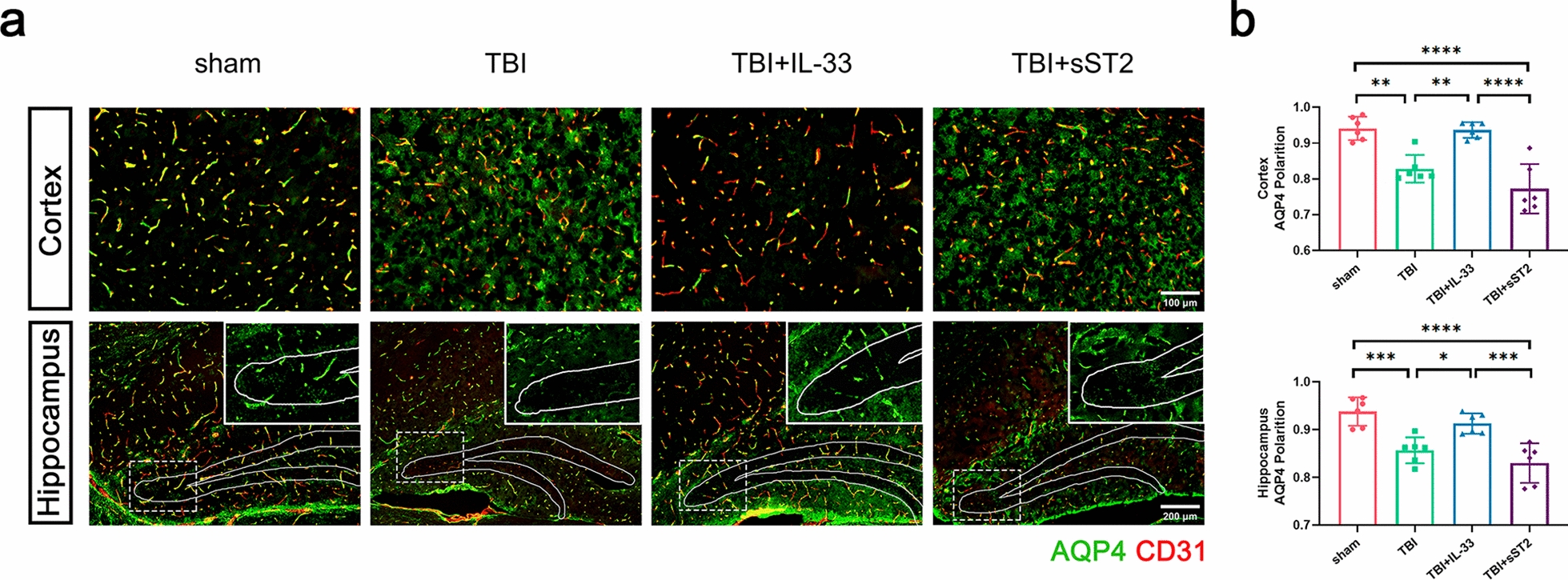
AQP4 polarization analysis in the region of capillaries of acute TBI mice. **a** Brain sections stained for CD31 (red) and AQP4 (green), illustrating AQP4 localization in the region of capillaries at the cortex (above) and hippocampus (below). Inset depicts AQP4 immunoreactivity alone. **b** Quantification of AQP4 polarization in the cortex (above) and the dentate gyrus of hippocampus (below). All data are presented as mean ± SD (n = 6 per group). *p < 0.05, **p < 0.01, ***p < 0.001, ****p < 0.0001

Considering that fluorescence images of the capillaries cannot distinguish the vessel lumen/wall, we used a different method to analyze the distribution of AQP4 in the microvessels (diameter < 10 μm) of the cortex and hippocampus of acute TBI mice by calculating the polarization of AQP4. After TBI, AQP4 fluorescence in the cortex was more dispersed and no longer localized explicitly along the capillaries but was more distributed in the brain parenchyma. Similar depolarization of AQP4 was observed in the dentate gyrus of the hippocampus, implying that the distribution of AQP4 no longer formed a vascular morphology and could not merge with CD31-labeled capillaries (Fig. 4a). Exogenous IL-33 significantly reversed this effect, and no significant difference was observed between the sST2 treatment and non-treatment groups. AQP4 polarization analysis also verified the fluorescence staining results (Fig. 4b).

### Exogenous IL-33 improved the drainage and promoted the proliferation of MLVs in acute TBI mice

**Fig. 5:**
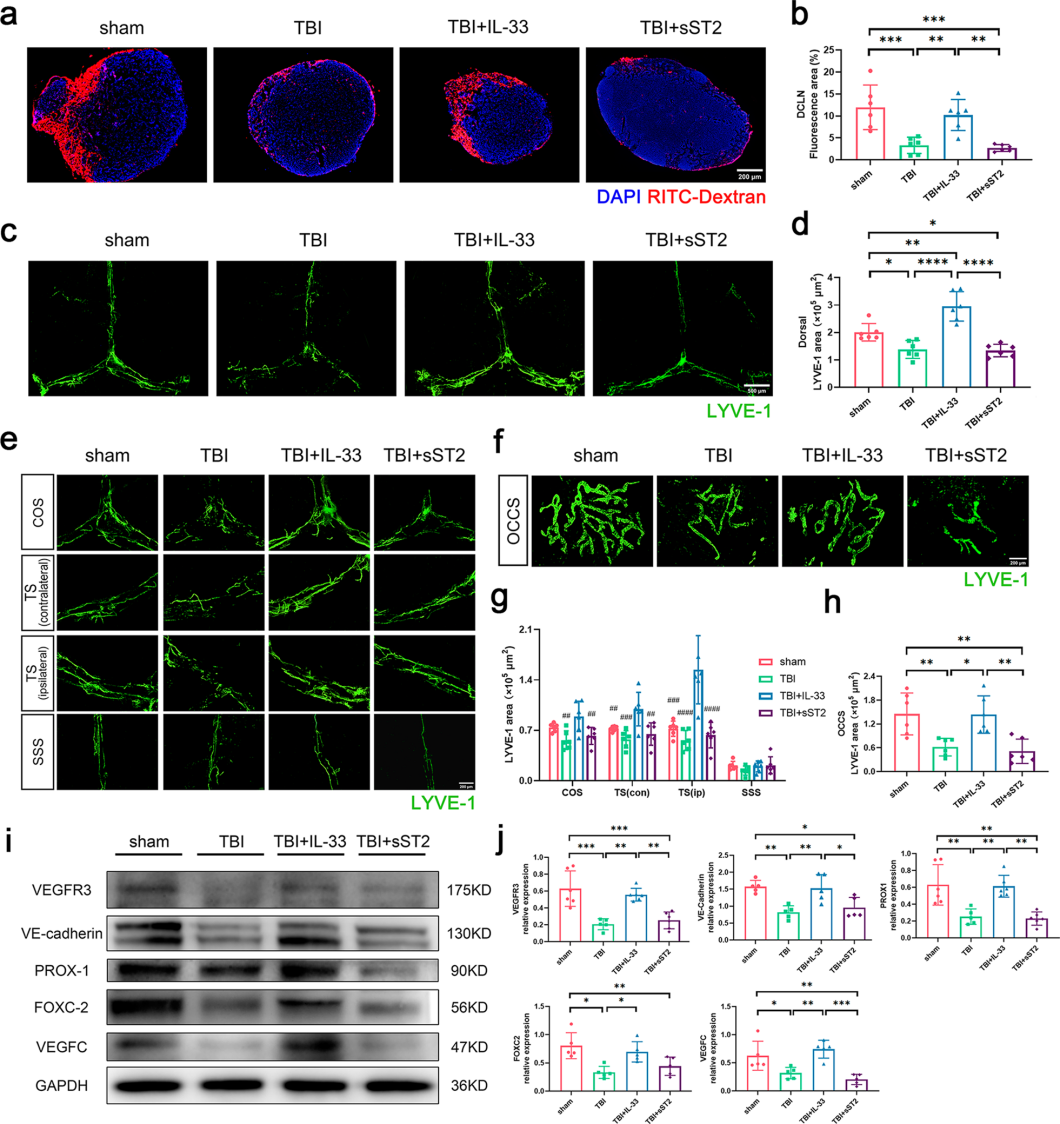
Changes of meningeal lymphatic drainage and related proteins of acute TBI mice. **a** Representative images of dCLN showing drainage of RITC-Dextran. **b** Graph depicting percent area of RITC-Dextran coverage of dCLN. **c** Representative immunofluorescence images of dorsal MLVs. **d** Quantification of LYVE-1 staining area of dorsal meninges. **e**–**f** Representative images of the area of LYVE-1 staining in dorsal COS, TS (contralateral), TS (ipsilateral), SSS, and basal OCCS. **g**–**h** Quantification of LYVE-1 positive area of each part of dorsal meninges and OCCS of basal meninges. **i**–**j** Western blot scans and quantitative analysis of VEGFR3, VE-cadherin, PROX-1, FOXC-2 and VEGFC protein in dorsal meninges. All data are presented as mean ± SD (n = 5–6 per group). *p < 0.05, **p < 0.01, ***p < 0.001, ****p < 0.0001. ##*p* < 0.01 versus TBI + IL-33 group, ###*p* < 0.001 versus TBI + IL-33 group, ####*p* < 0.0001 versus TBI + IL-33 group

The recently discovered dura meningeal lymphatics further connect to the dCLN, which are crucial for clearing macromolecules from the brain. To evaluate their function after TBI, we injected the tracer into the cisterna magna and sliced the dCLN 60 minutes later. We proved that the function of MLVs was undermined after TBI, with tracer drainage to the dCLN significantly reduced. The drainage in the IL-33 treatment group was significantly higher than that in the TBI and sST2 treatment groups (Fig. 5a–b).

The dorsal MLVs were reduced in density after TBI, and the LYVE-1 positive area was significantly decreased. The IL-33 treatment group was significantly increased in the LYVE-1^+^ area, which was higher than that in the non-treatment group and markedly higher than that in the sham group (Fig. 5c–d). The MLV coverage of the confluence of the sinus (COS), contralateral and ipsilateral sides of the transverse sinus (TS), and superior sagittal sinus (SSS) were calculated. It revealed that the MLV coverage of the IL-33 treatment group at COS and the contralateral and ipsilateral TS was significantly higher than that of non-treatment and sST2 treatment groups. Additionally, the enhancement of MLVs at TS was more apparent and even higher than that of the sham group. Particularly, the increase in MLV coverage on the ipsilateral side of the TS was more significant than that on the contralateral of the TS (Fig. 5e, g). Meninges at the basal occipital sinus (OCCS) were also collected and stained, and the MLVs at the OCCS showed similar intergroup changes in the dorsal area (Fig. 5f, h).

Western blotting analysis of dorsal meninges of mice demonstrated that protein VEGFR3, VE-cadherin, PROX-1, FOXC-2, and VEGFC, related to the proliferation and differentiation of MLVs and endothelial connection, decreased after injury while significantly increasing in the group treated with IL-33 in advance. No difference was observed between the sST2 treatment and non-treatment groups (Fig. 5i–j).

### Exogenous IL-33 reduced tau/p-tau accumulation and the inflammatory response in acute TBI mice

**Fig. 6:**
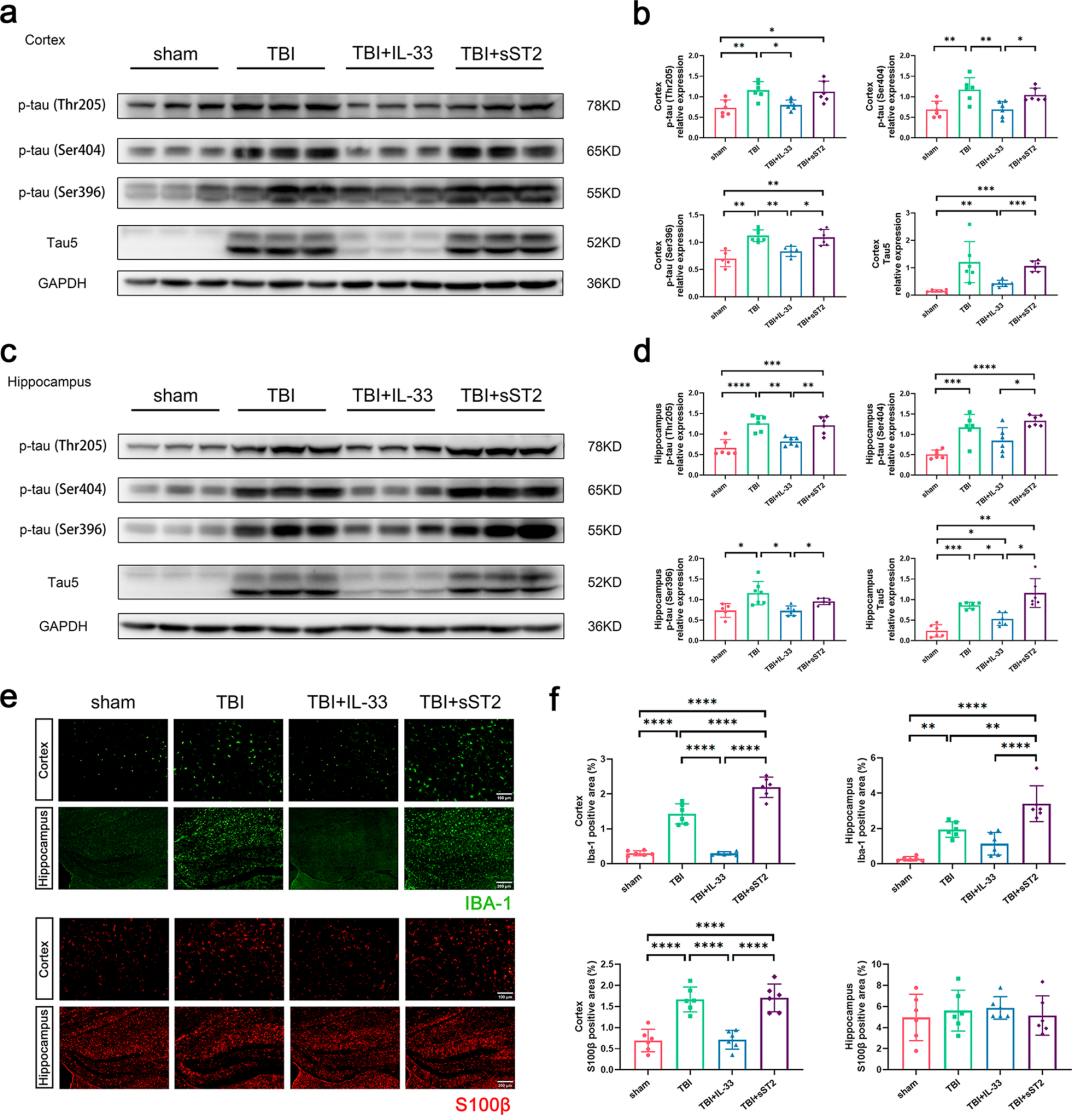
Tau5/p-tau accumulation and inflammatory response in the cortex and hippocampus of acute TBI mice. Western blot scans and quantitative analysis of tau5 and p-tau protein in the cortex (**a**, **b**) and hippocampus (**c**, **d**). **e**–**f** Representative images and quantification of the area of IBA-1 and S100β staining in the cortex and hippocampus. n = 5–7 per group. All data are presented as mean ± SD. *p < 0.05, **p < 0.01, ***p < 0.001, ****p < 0.0001

The accumulation of tau and p-tau after TBI has been previously reported [30]. To examine evidence of tau/p-tau accumulation in acute TBI mice, we detected the levels of tau and p-tau in the cortex (Fig. 6a) and hippocampus (Fig. 6c) 3 days after TBI by western blotting. The results showed that compared with the non-treatment and sST2 treatment groups, IL-33, the group’s levels of Tau5, p-tau (Thr205), p-tau (Ser404), and p-tau (Ser396) in the cortex and hippocampus were decreased. Specifically, statistically significant differences were observed in the levels of p-tau (Ser404) and p-tau (Ser396) levels in the cortex (Fig. 6b). While significant differences in Tau5, p-tau (Thr205), and p-tau (Ser396) levels were noted in the hippocampus (Fig. 6d).

It has been documented that MLV intervention can affect the brain’s inflammatory response in TBI [5]. We proved that in the acute phase after TBI, IBA-1 positive areas in mice’s cortex and hippocampus significantly increased, suggesting increased microglia activation, which was significantly decreased in the IL-33 treatment group. Moreover, microglia were more significantly activated in the sST2 treatment group than in the non-treatment group (Fig. 6e–f), indicating that IL-33 released after trauma also played a role. However, the activation of astrocytes was different. The analysis of cortical-activated S100^+^ astrocytes was similar to that of microglia in the non-treatment and IL-33 treatment groups. In contrast, no significant difference was observed between the sST2 treatment and non-treatment groups. Furthermore, no significant differences were found in astrocyte activation in the hippocampus among the four groups (Fig. 6e–f, bottom).

## Discussion

Current studies widely recognize that IL-33 is an important immune regulator, which plays a protective role in intracerebral brain hemorrhage [12, 21], stroke [74, 75], AD [42, 61], and other CNS diseases, and can alleviate disease-induced neurobehavioral deficits. Similar to previous results [22, 72], our experiment showed that exogenous IL-33 improved motor function, object memory, and spatial memory in TBI mice. However, previous studies have mainly discussed the regulatory role of IL-33 in immune inflammation. No study has analyzed the impact of IL-33 on TBI mice from the standpoint of regulating lymphatic drainage in the brain. Our previous studies (unpublished data) have shown that pre-resection or removal of CLN 1 day after injury to block intracranial drainage has different effects on the neurological prognosis of TBI mice. The motor and cognitive functions of mice with CLN excision 1 day after TBI were better than those with CLN pre-excision, suggesting that the brain’s drainage system plays a vital role in the acute stage after TBI. Therefore, we evaluated the effects of IL-33 on the glymphatic system and MLVs function in acute TBI mice.

The glymphatic drainage pathway is a perivascular spatial network discovered recently in rodent brains that promotes the exchange of CSF and interstitial fluid (ISF). It clears waste from the neuropil into meningeal and cervical lymphatic drainage vessels. In various pathological states, including aging [41], TBI [30], and AD [27, 62], the glymphatic system is weakened, affecting the clearance efficiency of harmful metabolites, such as Aβ and p-tau. Accumulation of Aβ plaques and neurofibrillary tangles of hyperphosphorylated tau are associated with cognitive decline in AD [66]. Through tracer drainage experiments, we demonstrated that exogenous IL-33 could improve the exchange efficiency of CSF and ISF in the cortex and hippocampus of mice with acute TBI. Therefore, we hypothesized that treating TBI mice with IL-33 could reduce the toxic metabolite deposition, thereby restoring many TBI-related functional changes; this was verified in subsequent experiments (see discussion below).

We further explored the molecular phenotypic changes in the glymphatic system affected by exogenous IL-33. AQP4 knockout impaired perivascular CSF-ISF exchange and interstitial Aβ clearance in an early description of the glymphatic system [31]. Recently, studies utilizing the AQP4-specific inhibitor TGN-020 [29] have reported that acute inhibition of AQP4 slows perivascular exchange and Aβ clearance [27, 60]. These results suggest that AQP4-mediated glymphatic transport plays a crucial role in the movement of perivascular fluid and solutes. AQP4 localization is lost in perivascular astrocytic endfeet and increases in non-perivascular fine processes during aging [68], post-trauma [30, 59], and ischemic brain [67, 68]. In patients with AD, the loss of perivascular AQP4 localization is implicated in an increased pathological burden of Aβ and p-tau and cognitive decline early in the illness [62]. Here, during the acute phase after TBI, cortical and hippocampal AQP4 proteins were elevated and diffused from the perivascular to the nonvascular brain parenchyma, accompanied by decreased penetrating vasculature tracer exchange. We demonstrated that exogenous IL-33 could reverse this situation, restoring AQP4 distribution in astrocyte endfeet and increasing the penetrating vasculature tracer exchange. Previous studies have demonstrated that reactive astrogliosis occurs in acute TBI mice, which may be linked to alterations in the expression levels of AQP4 protein and its polarization [30]. The polarization of AQP4 in astrocyte endfeet is thought to be dependent on the formation of orthogonal arrays of particles (OAPs) [65]. Recent studies have revealed that the assembly of perivascular OAPs is crucial for the anchoring of AQP4 at the astrocyte endfeet, and that AQP4 isoforms M23 and AQP4ex are involved in this process [14, 46, 54]. Furthermore, α-syntrophin is believed to be implicated in the polarized subcellular localization of perivascular AQP4 [7, 62]. Deletion of the α-syntrophin gene abolishes the perivascular astrocytic AQP4 localization, impairs CSF-ISF exchange, and contributes to neurodegeneration [62]. The impact of IL-33 on AQP4 expression levels and distribution may be due to its potential to inhibit reactive astrogliosis or stabilize AQP4 localization in the perivascular astrocytic endfeet by modulating AQP4 subtypes or membrane complex elements, including but not limited to α-syntrophin. However, further investigation is necessary to elucidate this mechanism.

The MLV system is part of the brain’s lymphatic drainage, a bridge between the brain parenchymal glymphatic system and CLN, and is involved in solute drainage. Patel et al. [56] reported that tau clearance was impaired in K14-VEGFR3-Ig transgenic mice, which lack a functional CNS lymphatic system. Recent studies have demonstrated that in closed TBI [5], subarachnoid hemorrhage [11], subdural hematoma [47], and cranial hypertension [71] models, the drainage function of MLVs is damaged, and the drainage of substances (hemocytes, among others) to the dCLN is restricted. Consistent with these findings, we also demonstrated impaired drainage of MLVs in TBI mice with reduced MLV density in the COS and TS. Our previous studies [71] and Louveau et al. [5] found that the tracer injected into the cisterna magna accumulated rapidly in the lymphatic vessels along the TS of the dura mater, suggesting that the MLVs of the TS is the main drainage pathway for dorsal MLVs. Here, we revealed that exogenous IL-33 could significantly increase lymphatic vessel density at the COS and TS, particularly the ipsilateral TS. This finding may explain why exogenous IL-33 improves the drainage of MLVs in mice with acute TBI; however, direct tracer aggregation evidence is still lacking.

The features of basal MLVs are distinct from those of the dorsal MLVs. These lymphatic capillaries are blunt-ended, with a button-like junctional pattern and lymphatic valves that facilitate CSF uptake and drainage [1]. Jacob et al. used light-sheet fluorescence microscopy imaging technology to fully demonstrate the presence of abundant lymphatics at the skull base. They presented direct evidence that the CSF tracer is carried out of the skull by the lymphatics at the skull base [35]. We found that the exogenous IL-33 also affected the density of lymphatic vessels at the OCCS; however, its effect on basal MLVs at other sites requires further investigation.

Various proteins regulate MLV proliferation and germination. The VEFRC/VEGFR3 signaling pathway plays a crucial role in the growth and migration of lymphatic endothelial cells [49]. PROX-1, FOXC-2, and VE-cadherin are believed to be involved in lymphatic vessel differentiation, development, and endothelial junction integrity, respectively [3, 53, 69]. Han et al. [26] showed that IL-33/ST2 signaling promotes the proliferation, migration, and tube formation of lymphatic endothelial cells in vitro and inflammation-induced lymphangiogenesis in vivo. Here, we suggest that exogenous IL-33 plays a role in improving the function of MLVs by upregulating the expression of the aforementioned lymphovascular-related proteins and promoting the proliferation and sprouting of MLVs.

Currently, it is believed that the cerebral drainage system can regulate the inflammatory response by managing the clearance of waste and pro-inflammatory cytokines. Ligation of dCLN in AQP4^−/−^ mice exacerbates microglial activation and hippocampal neuronal apoptosis in their brains and impairs cognition and exploration ability compared with wild-type mice [8]. In a mouse model of AD, ablation of MLVs exacerbates Aβ deposition, neurovascular dysfunction, microgliosis, and behavioral deficits. Additionally, microglia transform into a more inflammatory phenotype [13]. Restoration of MLV drainage in aged mice ameliorates TBI-induced microgliosis [5]. These findings suggest a close connection between changes in the brain’s drainage system and the regulation of inflammatory responses. Our results also support this link: TBI mice treated with exogenous IL-33 showed restored brain drainage function; the accumulation of tau/p-tau in the cortex and hippocampus and the activation of cortical and hippocampal microglia and cortical astrocytes were reduced.

Conversely, the IL-33/ST2 signaling pathway directly affects the neuroinflammatory response after CNS injury. Several studies have shown that IL-33 can play an anti-inflammatory role by promoting the polarization of microglia to the anti-inflammatory M2 type [12, 19, 58] and Treg expansion and activation [34, 72], suppressing astrogliosis and potentiating neurological recovery. We found that neutralizing endogenous IL-33 with sST2 resulted in significantly higher activation of cortical microglia in TBI mice than in untreated mice. Existing evidence supports a relationship between glymphatic function and gliacytes in the inflammatory state. Reactive astrogliosis and microglial morphological changes resulting from an inflammatory insult contribute to reduced brain CSF/ISF exchange and fluid transport [37, 50, 55]. Therefore, exogenous IL-33 may also reduce the activation of microglia and astrocytes through anti-inflammation, thereby increasing brain clearance, reducing tau/p-tau accumulation in the brain, and restoring neurological deficits.

Surprisingly, the neutralization of post-traumatically released IL-33 with sST2 did not significantly impair the brain’s lymphatic drainage and neurological function. This may be because of the following reasons: 1. Although no sufficient reports are available, IL-33 released into the CSF after spinal cord injury does not exceed 400 pg/μl in vitro and in vivo experiments [19], which is significantly less than the dose we provided (20 ng/μl). Therefore, the concentration of IL-33 released after TBI may not be sufficient to affect the brain’s lymphatic drainage; thus, no significant difference was found between the sST2 treatment group and the non-treatment group; 2. Blocking the IL-33/ST2 signaling pathway may activate other compensatory mechanisms to prevent brain drainage and neurological deterioration; 3. The evaluation method we used should be more sophisticated to show the difference between the blocking IL-33/ST2 signaling group and the non-treatment group in brain lymphatic drainage and neurological function; 4. In addition to neutralizing free IL-33, the sST2 protein can also play an independent role. Nagata et al. [51] reported that sST2 attenuates the intracellular signal transduction of lipopolysaccharide, subsequently, the pro-inflammatory cytokine production. sST2 may have an additional role in preventing the deterioration of lymphatic drainage and neurological function in the brain. Summarily, the in-depth mechanism of the role of the IL-33/ST2 signaling pathway in lymphatic drainage of the brain is still ambiguous, and the above hypothesis still needs to be verified by further experiments.

Our study has elucidated a distinct influence of extrinsic IL-33 on the glymphatic system and MLVs. Nonetheless, it is imperative to acknowledge that the intramural periarterial drainage (IPAD) pathway, characterized by the flow of ISF through the basement membrane in the walls of cerebral capillaries and arteries, is also regarded as a significant drainage route for the brain [9]. The contraction of smooth muscle cells primarily regulates this pathway [2, 16]. In addition, this pathway can clear various brain metabolites, such as Aβ, tau, and α-synuclein protein [28, 52]. The changes that occur in this pathway following TBI and the effect of exogenous IL-33 on this pathway necessitate further investigation.

## Conclusion

Our study is the first to discuss the protective effect of IL-33 on TBI mice from the perspective of regulating brain lymphatic drainage, demonstrating that exogenous IL-33 can enhance CSF-ISF exchange in the cortex and hippocampus in the acute phase of TBI mice, reverse the dysregulation and depolarization of AQP4, and improve the drainage of MLVs. We believe that the enhancement of cerebral lymphatic drainage and toxic metabolite clearance by exogenous IL-33 is related to improving neurological function in TBI mice. Additionally, we speculated that an interaction exists between the activation of gliacytes induced by inflammatory responses and the brain’s drainage function under the intervention of IL-33. Combined with the reported results, these data further support the notion that IL-33 therapy may be an effective therapeutic strategy for alleviating acute brain injury after TBI.

## Data Availability

The datasets used and/or analysed during the current study available from the corresponding author on reasonable request.

## Abbreviations

TBI: Traumatic brain injury
MLV: Meningeal lymphatic vessel
IL-33: Interleukin 33
AD: Alzheimer’s disease
AQP4: Aquaporin-4
VEGFC: Vascular endothelial growth factor C
CNS: Central nervous system
MS: Multiple sclerosis
CLN: Cervical lymph nodes
FPI: Fluid percussion injury
RITC-Dextran: Rhodamine B isothiocyanate dextran
CSF: Cerebrospinal fluid
ISF: Interstitial fluid
COS: The confluence of the sinus
TS: Transverse sinus
OCCS: Occipital sinus
SSS: Superior sagittal sinus
OAPs: Orthogonal arrays of particles
IPAD: Intramural periarterial drainage
